# Nicotinamide adenine dinucleotide reduced (NADH) is a natural UV filter of certain bird lens

**DOI:** 10.1038/s41598-022-21139-x

**Published:** 2022-10-07

**Authors:** Nataliya A. Osik, Ekaterina A. Zelentsova, Kirill A. Sharshov, Yuri P. Tsentalovich

**Affiliations:** 1 International Tomography Center SB RAS, Institutskaya 3a, Novosibirsk, 630090 Russia; 2 Federal Research Center of Fundamental and Translational Medicine, Timakova 2, Novosibirsk, 630117 Russia

**Keywords:** Metabolomics, Biophysical chemistry

## Abstract

In this work, we for the first time report the identification of UV filters in the bird eye lens. We found that lenses of some raptors (black kite, common buzzard) and waterfowl (birds from Podicipedidae family) contain unusually high levels of reduced nicotinamide adenine dinucleotide (NADH)—a compound with high absorption in the UV-A range with a maximum at 340 nm. The lens metabolome of these birds also features an extremely low [NAD +]/[NADH] ratio. Chemometric analysis demonstrates that the differences between the metabolomic compositions of lenses with low and high NADH abundances should be attributed to the taxonomic features of bird species rather to the influence of the low [NAD +]/[NADH] ratio. We attributed this observation to the low metabolic activity in lens fiber cells, which make up the bulk of the lens tissue. Photochemical measurements show that properties of NADH as a UV filter are as good as that of UV filters in the human lens, including strong absorption in the UV-A spectral region, high photostability under both aerobic and anaerobic conditions, low yields of triplet state, fluorescence, and radicals under irradiation. Lenticular UV filters protect the retina and the lens from photo-induced damages and improve the visual acuity by reducing chromatic aberrations; therefore, the results obtained contribute to our understanding of the extremely high acuity of the raptor vision.

## Introduction

The bird visual system is considered as one of the most acute among all animals. The safety of a flight, successful hunting, and subsistence of birds are almost completely ensured by the acute vision. A bird eye has a number of adaptations maximizing the visual acuity. In particular, birds have a large eye in relation to the size of the head and the whole body. Ciliary muscles of the bird eye change the lens shape faster and stronger than that in mammals. The density of photoreceptors in the bird retina (especially in the retina of raptors) is significantly higher than that in other animals^1,2^, and the presence of a special organ, pecten oculi^3,4^, also greatly contributes to the sensitivity of the vision system, reducing the number of blood vessels in the retina.

The majority of birds have tetrachromatic vision: there are four types of cone cells in the retina with the different spectral ranges, which improves the color differentiation of the vision. In particular, many birds are able to see UV light^5,6^. However, the penetration of UV light deep into the ocular system may present serious disadvantages. Firstly, high-energy quanta of UV light may initiate undesirable photochemical reactions and cause light-induced damage to the retina and eye lens. Secondly, a broad spectrum of light reaching the retina causes chromatic aberrations related to different refractive indexes of the lens at different wavelengths. Due to the inverse dependence of the refractive index on the wavelength, UV light makes the greatest contribution into the retinal image distortion. The ocular media of majority of birds is partly transparent for UV-A (315–400 nm) light. However, Olsson et al.^7^ showed that for some bird species, including birds of prey and waterfowl, the ocular media transmittance is limited by the lens and corneal absorbance at wavelengths below 400 nm. This may indicate the presence in the bird lens of UV-absorbing pigments reducing chromatic aberration, improving bird visual acuity, and protecting the retina and the lens from the harmful UV radiation.

Molecular UV filters absorbing UV-A radiation were first identified in the human lens^8^. These compounds are kynurenine (KN) and its derivatives (3-hydroxykynurenine, 3-hydroxykynurenine O-β-d-glucoside, and others)^9–12^. Acting as natural UV filters, kynurenines protect retinal cells from UV light and prevent photoinduced damages of lens proteins. Besides humans and other primates^13–15^, UV filters have so far been found in only two species of squirrels^15–17^ among all terrestrial vertebrates. Among other animals, the lenticular absorption in UV-A region was reported for fish, snakes, and amphibians^18–22^. However, the identification of molecular UV filters was performed only for some fish species.

**Figure 1:**
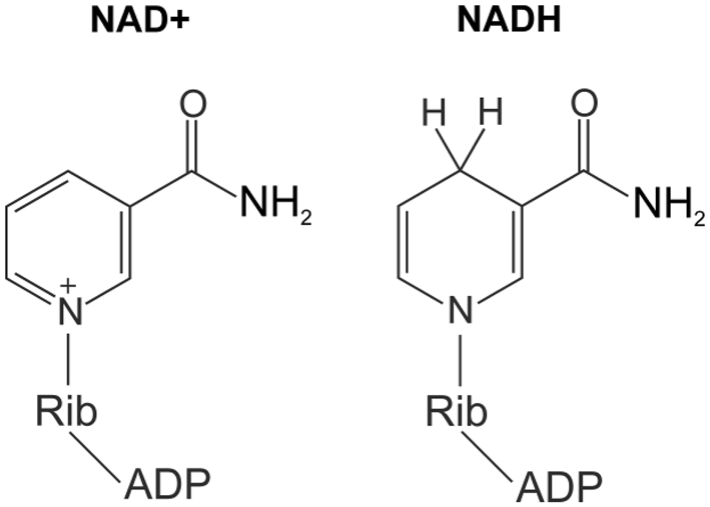
Structures of NAD+ and NADH.

In our recent work^23^, we studied the metabolomic composition of lenses from 14 bird species, and detected unexpectedly high levels of NADH (β-Nicotinamide adenine dinucleotide reduced, Fig. 1) in lenses of two species, black kite (*Milvus migrans*) and great crested grebe (*Podiceps cristatus*). NADH is known to absorb in the UV-A region^24^, so this compound can be considered as a potential UV filter of the bird lens. In the present work, we attempted to perform a directed search of bird species, whose lenses may contain UV filters. We paid special attention to diurnal birds of prey and waterfowl: these birds need an excellent eyesight for hunting, while the eye is exposed to the sun radiation for a long time. The major goals of the study were to determine the bird species whose lenses contain UV filters, to identify these compounds, and to characterize their photochemical properties. To this end, we performed quantitative metabolomic analysis of the lens tissues of several bird species. We also measured the absorbance of the bird lens metabolites and studied the photochemical properties of the potential UV filters.

## Results

### Quantitative metabolomic analysis of bird lenses

**Figure 2:**
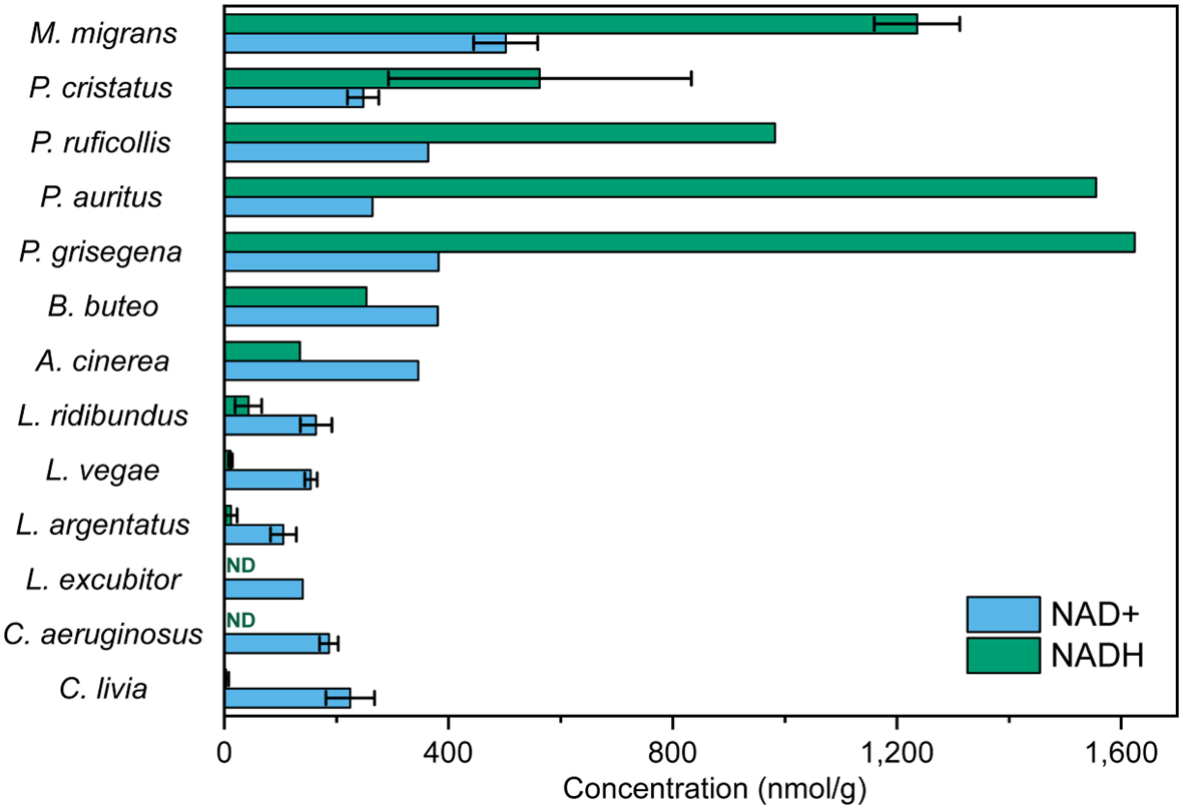
Levels of NADH and NAD + in the bird lenses (in units of nmol per gram of the wet tissue). *ND* not detected.

In the present work the lens metabolomic composition of 13 bird species was studied. The bird species and the number of individuals within each species used for metabolomic analysis as well as the territorial localization of the samples collection are shown in Table 1. NMR-based metabolomic analysis of bird lenses was performed in the same way as described in^23^, and the complete set of obtained results is presented in Table S1, where the metabolite concentrations in lenses are expressed in units of nmol per gram of the wet tissue. The data for *M. migrans, P. cristatus,* and *C. livia* are taken from^23^. The data on reduced NADH and oxidized NAD + forms of nicotinamide adenine dinucleotide are collected in a separate table (Table 2). High lenticular levels of NADH were found for all four species from the *Podiceps* genus (varying from 400 to 1650 nmol g^−1^) and for *M. migrans* (1200 nmol g^−1^). The concentration of NADH in lenses of these birds is 2–5 times greater than that of NAD+. In lenses of *C. aeruginosus* and *L. excubitor*, the concentration of NADH was below the measurable level (which in our case can be estimated as approximately 5–10 nmol g^−1^). The lenses of *B. buteo* and *A. cinerea* occupy an intermediate position: the NADH level is relatively high (250 nmol g^−1^ and 140 nmol g^−1^ respectively), but it is lower than the concentration of NAD+ (380 nmol g^−1^ and 350 nmol g^−1^). Figure 2 shows the graphical presentation of NADH and NAD + levels in lenses of the birds under study.

**Table 1:** Description of the bird species used for the lens metabolomic analysis.

Bird species	Place of the sample collection	Number of individuals	Lens weight (mg)
Black kite (*Milvus migrans*)	Tyva Republic (July 2019)	5	90–158
Great crested grebe (*Podiceps cristatus*)	Tyva Republic (May 2019)	5	67–83
Little grebe (*Podiceps ruficollis*)	Tyva Republic (May 2019)	1	47.0
Horned grebe (*Podiceps auritus*)	Novosibirsk region (September 2019)	1	72.1
Red-necked grebe (*Podiceps grisegena*)	Novosibirsk region (September 2019)	1	42.9
Common buzzard (*Buteo buteo*)	Novosibirsk region (July 2019)	1	204.6
Grey heron (*Ardea cinerea*)	Novosibirsk region (September 2020)	1	350
Black-headed gull (*Larus ridibundus*)	Tyva Republic (May 2019)	7	62–99
Vega gull (*Larus vegae*)	Tyva Republic (May 2019)	3	135–153
European herring gull (*Larus argentatus*)	Tyva Republic (July 2019)	5	79–112
Great grey shrike (*Lanius excubitor*)	Novosibirsk region (August 2019)	1	47.6
Western marsh harrier (*Circus aeruginosus*)	Dagestan Republic (September 2021)	4	80–131
Rock dove (*Columba livia*)	Novosibirsk region (September 2017)	12	25–56

**Table 2:** Concentrations of NADH and NAD + in the bird lenses and the ratio of mean values [NAD +]/[NADH].

Bird species	[NADH], nmol g^−1^	[NAD +], nmol g^−1^	[NAD +]/[NADH]
Black kite (*M. migrans*)	1240 ± 80	500 ± 60	0.40
Great-crested grebe (*P. cristatus*)	560 ± 270	248 ± 28	0.44
Little grebe (*P. ruficollis*)	982	364	0.37
Horned grebe (*P. auritus*)	1555	264	0.17
Red-necked grebe (*P. grisegena*)	1624	382	0.24
Common buzzard (*B. buteo*)	254	381	1.50
Grey heron (*A. cinerea*)	135	346	2.60
Black-headed gull (*L. ridibundus*)	40 ± 28	140 ± 60	3.5
Vega gull (*L. vegae*)	11 ± 3	154 ± 11	14.0
European herring gull (*L. argentatus*)	12 ± 11	105 ± 23	8.8
Great grey shrike (*L. excubitor*)	ND	140	-
Western marsh harrier (*C. aeruginosus*)	ND	186 ± 17	-
Rock dove (*C. livia*)	3 ± 5	220 ± 40	73

*ND* not detected.

**Figure 3:**
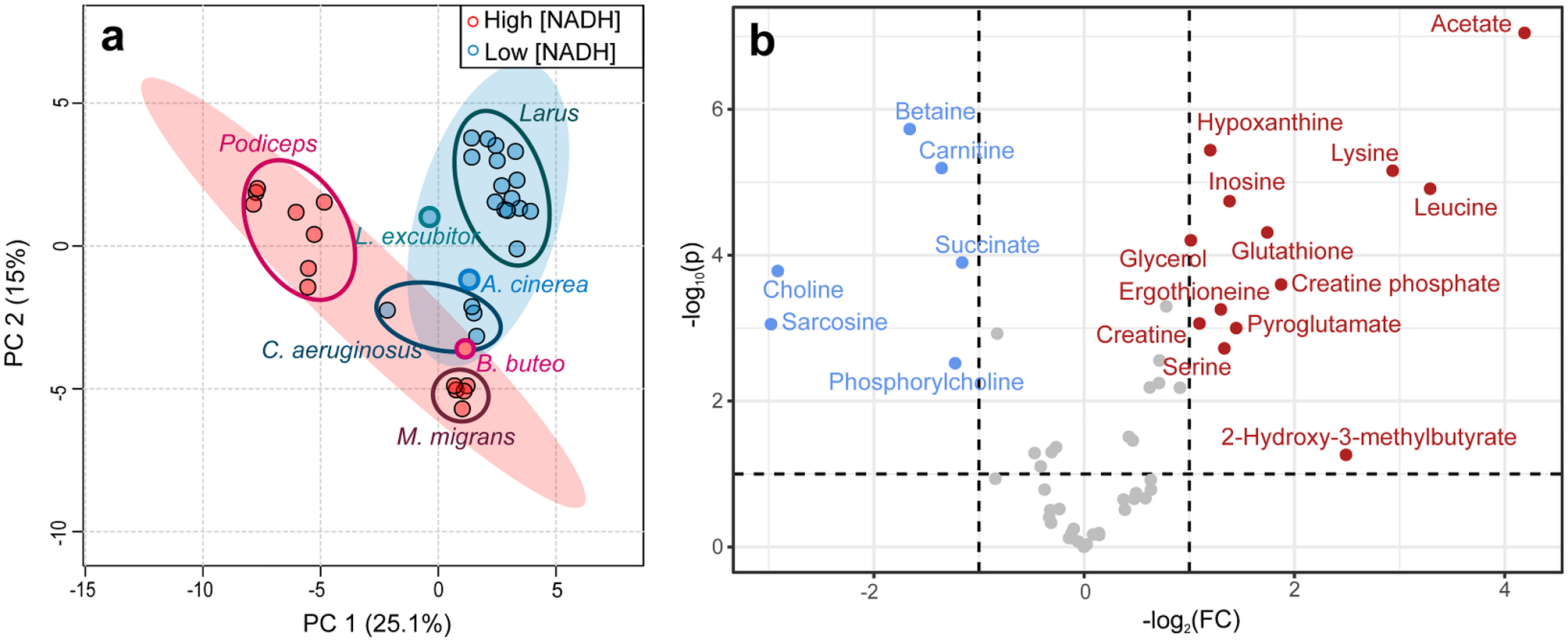
(**a**) Scores plot of principal component analysis (PCA) of bird lens metabolomic profiles for the groups of bird species with high and low NADH content in the lens. Filled colored ovals indicate 95% confidence regions; (**b**) Volcano plot for metabolites corresponding to the high and low NADH levels. Red and blue dots indicate significant increase (p < 0.1, FC > 2) and decrease (p < 0.1, FC < 0.5) of concentrations in the group with high NADH content, respectively.

NADH is an important coenzyme; it participates in a number of biochemical reactions and metabolic cycles^25^, and its high level in the eye lens may affect the general metabolomic composition of the lens tissue. To check this hypothesis, we performed the principal component analysis (PCA) for two group of samples: lenses of birds of prey and waterfowl with the high (> 200 nmol g^−1^) NADH content and with the low one (Fig. 3a). During the analysis, we removed NAD + and NADH from the metabolite list to avoid their direct influence on the principal component formation. Figure 3a shows that the samples corresponding to the same bird species form distinct clusters, demonstrating a major role of the genetic factor on the lens metabolomic composition. Two groups of species with the high and low NADH levels are separated, but their confidence regions overlap. This probably indicates the correlation between the NADH levels and the total lens metabolomic composition, but this correlation seems to be weak as compared with the taxonomy factor.

The results of the hierarchical clustering analysis (HCA) confirm this conclusion. HCA dendrogram (Fig. S1) was constructed with the use of Euclidian distance and Ward’s linkage for auto-scaled data. It demonstrates that the samples belonging to the same species group in separate clusters, and the position of species in the dendrogram are determined mostly by their taxonomy.

Figure 3b shows the Volcano plot for metabolites corresponding to the high and low NADH levels. It demonstrates that the statistically significant increase (p < 0.1, FC > 2) for lenses with the high NADH level are observed for 13 compounds (the highest difference was found for acetate, lysine, and leucine), and the statistically significant decrease (p < 0.1, FC < 0.5)—for 7 compounds (the highest difference for choline and sarcosine).

### Optical study of the bird lens metabolome

**Figure 4:**
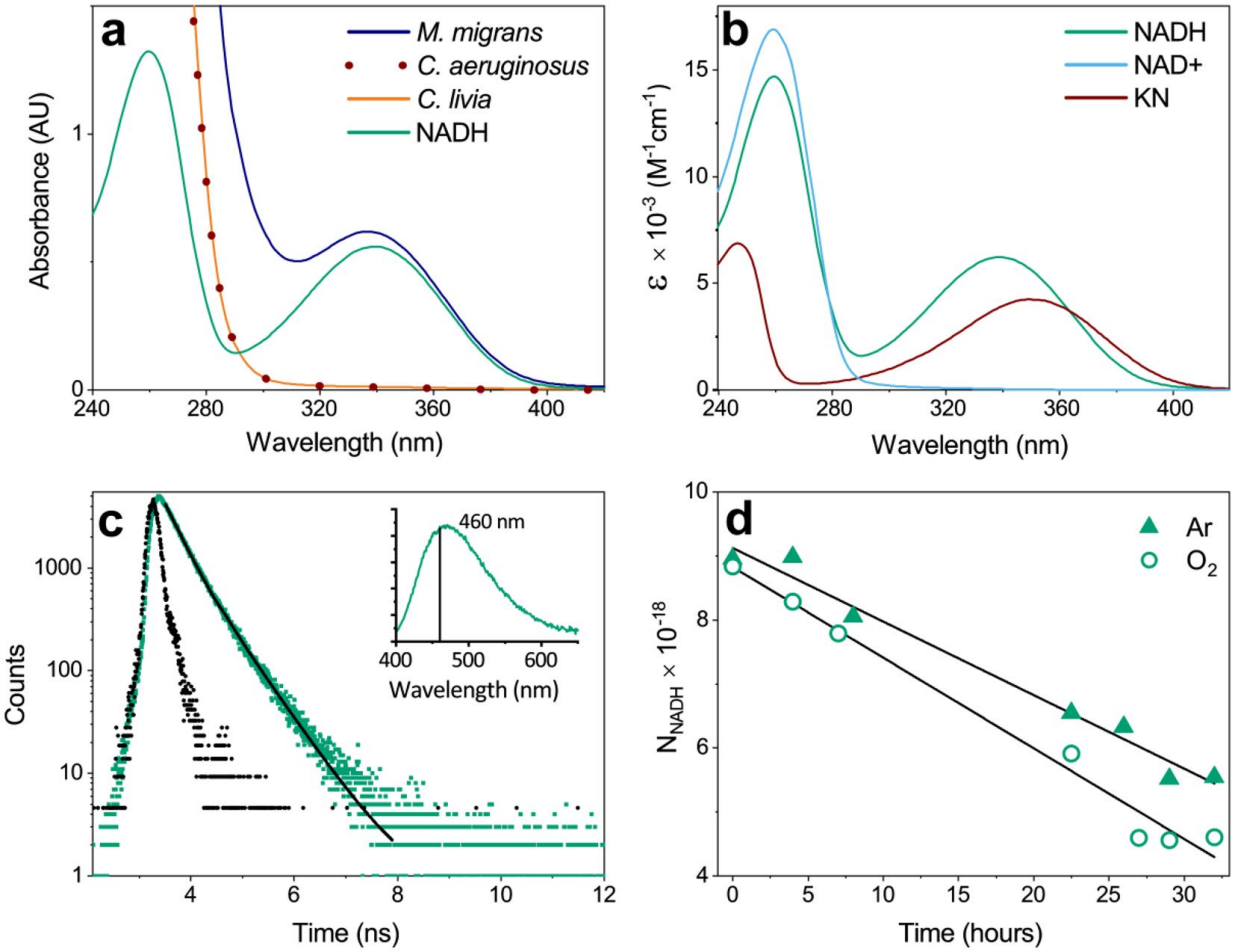
(**a**) UV–Visible spectra of bird lens metabolomic extracts in aqueous solution and the spectrum of 0.08 mM NADH aqueous solution; (**b**) Optical absorption spectra of NADH, NAD + and KN; (**c**) Kinetics of NADH fluorescence decay (green dots), measured at λ_em_ = 460 nm, convolved with IRF (black dots) and fitted by a biexponential function (black line); insert: a steady-state fluorescence spectrum of NADH; (**d**) Kinetics of NADH photodecomposition (in units of number of molecules in 3 mL of aqueous solution) during 300–400 nm photolysis under Ar and O_2_ with the correction on the solvent evaporation during the experiment.

For spectrophotometric measurements, we dissolved dry metabolomic extracts from lenses of *M. migrans*, *C. livia*, and *C. aeruginosus* in PBS, and recorded UV–Visible absorption spectra (Fig. 4a). The metabolomic fraction of the *M. migrans* lens has a strong absorption band between 300 and 400 nm, while metabolites from lenses of *C. livia* and *C. aeruginosus* do not absorb light in the UV-A region. The absorption spectrum of the extract from the *M. migrans* lens in the 300–400 nm region practically coincides with the spectrum of NADH, indicating that this compound is the only chromophore among lenticular metabolites of *M. migrans*.

### Photochemical study of NADH

The NADH UV–Visible spectrum (Fig. 4b) has two absorption maxima at 260 nm and 340 nm. The latter corresponds to the absorption of the nicotinamide residue^24,26^. Figure 4c shows the fluorescence spectrum obtained under 340 nm steady-state irradiation of 4 × 10^–5^ M NADH aqueous solution, and the fluorescence kinetics measured at the emission maximum with the excitation wavelength of 374.6 nm. The fluorescence decay is well fitted by a biexponential function with the characteristic times of 0.24 ± 0.04 ns (A ≈ 0.12) and 0.604 ± 0.010 ns (B ≈ 0.05). These components correspond to cis and trans configurations of the nicotinamide ring^27,28^. The obtained spectral and kinetic data on NADH absorption and fluorescence are in a good agreement with previous reports^27–30^.

We studied photoinduced reactivity of NADH by means of LFP. 2 × 10^–4^ M NADH aqueous solution placed into a photochemical cuvette was irradiated by 355 nm laser pulses; the optical density of the solution at 355 nm was OD = 0.93. With the laser energy below 50 mJ per pulse, no signals of transient absorption have been detected in the spectral range from 280 to 680 nm. The increase of the laser energy above 50 mJ/pulse resulted in the appearance of a decaying absorption band at the long-wavelength (above 450 nm) region accompanied by a negative transient signal at the wavelengths of NADH absorption with the maximum at 340 nm. The intensities of both positive signal at the long wavelengths and negative signal at the short wavelengths increased quadratically with the laser energy increase. We suggest that the observed optical changes correspond to biphotonic NADH ionization with the positive signal corresponding to solvated electron and the negative signal—to the depletion of the starting compound. To check this assumption, we added a well-known electron scavenger acetone^31^ into the cuvette in the concentration of 35 mM. This resulted in complete disappearance of the positive signal at the long wavelengths, while the negative signal remained. Acetone scavenges solvated electrons with the rate constant of 6 × 10^9^ M^−1^ s^−1^^32^ with the formation of an optically silent adduct, and the observed disappearance of the positive signal confirms the solvated electron formation. The biphotonic ionization of NADH is in a good agreement with earlier published data^33–35^. At the same time, the obtained results indicate that monophotonic reactions of NADH do not yield detectable amounts of triplet state or radicals.

To estimate the NADH photostability, we performed steady-state photolysis of NADH aqueous solution under aerobic and anaerobic conditions. The NADH concentration of 5 mM provided an almost complete absorbance of UV light in the solution in the 300–400 nm spectral region. The kinetics of the photodecomposition was measured by ^1^H NMR spectroscopy (Fig. 4d). After 32 h of NADH exposure to UV light, the initial compound decomposed by about 40–50% under both aerobic and anaerobic conditions. We should note that the gas bubbling through the solution during a long time resulted in a partial solvent evaporation (approximately 10% for 32 h of bubbling). This effect was taken into account in the calculations of the decomposition quantum yield. HPLC–MS and NMR analyses of the irradiated solutions showed that the main products of the photodecomposition are NAD + and adenosine diphosphate ribose, which is in line with the results of Vitinius et al.^36^. The calculated quantum yields of NADH photodecomposition are (1.9 ± 0.3) × 10^–5^ and (2.3 ± 0.3) × 10^–5^ under anaerobic and aerobic conditions, respectively.

## Discussion

Unusually high NADH concentrations in lenses of *M. migrans*, *B. buteo*, and podicipediformes indicate that this substance can be considered as a potential UV filter of the bird lens. NADH has a strong absorption band with the maximum at 340 nm and molar extinction of 6300 M^−1^ cm^−1^. The metabolomic extract of the *M. migrans* lens exhibits a pattern similar to the shape of the NADH absorption in the range of 300–400 nm (Fig. 4b). We can estimate that for the *M. migrans* lens with the typical diameter of 0.6 cm and NADH concentration of 1200 nmol g^−1^, the optical density at 340 nm is approximately 4.5, i.e. the attenuation of UV light may exceed four orders of magnitude. Thus, NADH was proved of being the main UV-A chromophore in the lens of *M. migrans*, *B. buteo*, and all four species of *Podiceps* genus studied in this work. It is noteworthy that we also found unusually high concentration (113 nmol g^−1^) of NADPH in the lens of *A. cinerea*. Since NADPH as well as NADH absorbs in the UV-A region^37^, this may indicate that some birds probably use NADPH as UV filter in addition to NADH.

Lenticular UV filters are not common for the animal kingdom. So far, they were reported only for lenses of several mammals: human^8,10,12,38^, macaque^13^, grey^16^ and thirteen lined ground^17^ squirrels. In mammalian lenses, the UV-filtering function is fulfilled by kynurenine (KN) and its derivatives: 3-hydroxykynurenine O*-*β-D-glucoside (the most abundant UV filter in the lens of primates), N-acetylkynurenine and N-acetyl-3-hydroxykynurenine (main UV-filters in the lens of squirrels), 3-hydroxykynurenine, and others. The synthesis of these compounds occurs in the lens epithelial cells from amino acid tryptophan via relatively complex enzymatic mechanisms^9,39,40^, and then the synthesized compounds diffuse toward the lens core^41^. It seems that for the bird lens, nature has chosen a simpler way by using the much more common compound NADH as the UV filter.

Due to the biological importance of NADH, its chemical properties have already been partially studied^24,29,30,33,35^. However, the role of NADH as a UV filter has not been reported previously. Besides absorbance in UV-A region, the main requirements for UV-filtering pigments are high photochemical stability, low yields of reactive photoproducts and irradiative channels of relaxation after photoexcitation. The results of the present and previous^24,30,36^ works indicate that NADH meets all these requirements: the decomposition quantum yield is very low under both aerobic and anaerobic conditions, the formation of triplet states or radicals was not found in LFP experiments, and the main channel of the photo-excited state decay is the nonradiative transition to the ground state with the quantum yield of about 0.98. Photochemical properties of NADH can be compared with that of KN, the best-studied UV filter of the human lens^11,42–44^ (Table 3). The comparison shows that UV-filtering qualities of these compounds are similar: NADH has somewhat higher absorption coefficient and lower yields of the triplet state and aerobic photodecomposition, but the fluorescence yield for KN is lower.

**Table 3:** Photochemical parameters of NADH and KN: λ_max_ is the wavelength corresponding to the maximum optical absorption in the UV-A range, ε_max_ is the molar absorption coefficient at λ_max_, Φ_fl_ is the quantum yield of fluorescence, Φ_dec(Ar)_ and Φ_dec(O2)_ are the quantum yields of photodecomposition in an environment saturated with argon and oxygen, respectively.

	λ_max_ (nm)	ε_max_ (M^−1^ cm^−1^)	Φ_fl_ × 10^4^	Φ_T_ × 10^3^	Φ_dec(Ar)_ × 10^5^	Φ_dec(O2)_ × 10^5^
NADH	340	6300	20.0*	Not detected	1.9 ± 0.3	2.3 ± 0.4
KN**	360	4500	9.2 ± 1.0	7.0 ± 1.7	1.5 ± 0.2	11.0

*The value obtained by T.G. Scott et al.^30^.

**The data for KN obtained by Tsentalovich et al.^11,42,43^.

NADH is a vital one-electron carrier in animal cells. The NAD + /NADH redox couple is integrated into cellular metabolism through such processes as glycolysis, oxidative phosphorylation, and TCA cycle^25,45^. Free cytosolic NAD + /NADH ratio in mammalian cells has been reported to be up to 700, while in mitochondria this ratio is about 10^45,46^. In rat, human, and rabbit lenses the content of NAD + is several times higher than the content of NADH^47,48^. Our results demonstrate an inverse relationship between the concentrations of NAD + and NADH with a greater predominance of the latter in the lens tissue of some bird species. In the metabolically active tissues this imbalance would indicate disturbances in the metabolic cycles or even the pathologies in the tissue^49–51^. For example, nicotinamide adenine dinucleotide participates in the transformation of isocitrate into succinyl-CoA in the TCA cycle, and low [NAD +]/[NADH] ratio should result in a significant drop of the succinate and fumarate concentrations. However, in our measurements we did not observe any effect of NADH on fumarate level, and rather small effect for succinate (Fig. 3b and Table S1). We did find that the concentrations of several metabolites in the bird lenses with the high and low NADH levels differ, and it is tempting to attribute these differences to the influence of NADH on metabolic processes inside the lens. However, a careful inspection of the metabolomic data reveals that these differences correspond mostly to specific bird species rather than to the whole group “Lenses with the high NADH level”. For example, the concentration of acetate in lenses of *M. migrans* and *P. cristatus* is high, while in lenses of other birds from Podicipedidae family it is as low as in lenses of Laridae. The level of leucine is high in lenses of *Podiceps*, but it is relatively low in lenses of *M. migrans*, and so on. Therefore, we should conclude that the observed metabolomic differences correspond to the bird genotype and lifestyle rather than to the influence of high NADH concentration. It should be noted that the major part of the lens consists of metabolically inert fiber cells with the exception of metabolically active epithelial cells. We can assume that the direct influence of NADH on metabolic reactions and metabolomic composition might be noticeable for the epithelial monolayer and probably for a thin layer of the outer cortex, but for the whole lens, a large volume of fiber cells with minimal metabolic activity conceals this effect. For a better understanding of the effect of high NADH concentration on metabolic processes in living cells, it would be very interesting to compare the metabolomic compositions of the lens epithelial cells from a bird with high lenticular NADH abundance (such as black kite) with that from birds with low NADH level (such as doves or gulls).

All bird species studied in this work are diurnal animals; therefore, they need an accurate vision. The retina of diurnal raptors is equipped with four types of single cones with the short wavelength sensitivity at the violet range (VS)^2,6^. Notably, that cone photoreceptors contain special oil droplets, which are colorless for violet-sensitive cones and colored for three other ones. The pigmented oil droplets narrow the spectral sensitivity of cones and absorb most of UV light. Thus, UV light penetrating through the ocular media can affect only the VS receptors. However, rod cells responsible for the achromatic vision do not have additional UV protection. Therefore, one can hypothesize that filtering UV-A light by lenticular NADH has little effect for the color vision of birds, but may significantly improve the acuity of the achromatic vision due to reducing the chromatic aberrations.

It is worth mentioning that despite similar lifestyles and even relatively close genetic relationships, we found high NADH levels only in some raptors and waterfowl studied in this work. For example, both *M. migrans* and *C. aeruginosus* are birds of prey belonging to the same Accipitridae family, but the lens of the latter does not contain measurable amounts of NADH. Very likely, this phenomenon can be explained as the evolutional adaptation corresponding to the different ways of hunting: *M. migrans* looks out for prey from a great height, which requires extremely sharp vision, while *C. aeruginosus* hunts mainly from low height or from ambush. Among waterfowl, all grebes contain significant concentrations of NADH in the lens, while gulls do not. Within the *Podiceps* genus, the NADH level varies from 500 nmol g^−1^ (*P. cristatus*) to 1650 nmol g^−1^ (*P. grisegena*). Most likely, the genetic factor is responsible for the presence of NADH in the lens of certain bird species. It will be interesting to perform the metabolomic analysis for a larger group of birds, especially for ones with the low transmittance of the ocular media in UVA spectral range^7^, such as swan, swift, nightjar, or falcon. That may help to establish the relationship between the bird taxonomy and the presence of UV filters in their lenses. It is possible that a cornea can also contain pigments absorbing UV light; however, taking into account that the border of the cornea transmittance rarely exceeds 340 nm^7^, the cornea does not look like a promising media for the UV filter search.

## Materials and methods

### Chemicals

β-Nicotinamide adenine dinucleotide reduced was purchased from Serva (Heidelberg, Germany). Chloroform from Chimmed (Moscow, Russia), methanol from Merck (Darmstadt, Germany), D_2_O 99.9% from AstraChem (Saint-Petersburg, Russia) were used as received. H_2_O was deionized using an Ultra Clear UV plus TM water system (SG water, Hamburg, Germany) to the quality of 17.8 MOhm. Chemicals for HPLC were purchased from Sigma-Aldrich (St.Louis, MO, USA) and Cryochrom (Saint-Petersburg, Russia).

### Sample collection

The study was conducted in accordance with the ARVO Statement for the Use of Animals in Ophthalmic and Vision Research and the European Union Directive 2010/63/EU on the protection of animals used for scientific purposes, and with the ethical approval from the Ethics committee of International Tomography Center SB RAS (ECITC-2017-02). The study is reported in accordance with ARRIVE guidelines. Samples from the red-necked grebe (*Podiceps grisegena*), grey heron (*Ardea cinerea*), and great grey shrike (*Lanius excubitor*) were provided by the Center for the Rehabilitation of Wild Animals (CRWA, Novosibirsk) after the humane euthanasia of severely wounded bird. Other bird species of wild origin were collected during the hunting seasons 2017–2021 with a license from the regional Ministries of Ecology and Natural resources (Tyva Republic; Dagestan Republic; Novosibirsk Region) as part of the annual collection of biological material under the program for the study of infectious diseases of wild animals with approval of the Biomedical Ethics Committee of FRC FTM, Novosibirsk (Protocols No. 2013-23 and 2021-10). We extracted lenses from the body (usually within 1 h post mortem), froze in liquid nitrogen, and kept at − 70 °C until the analysis.

### Sample preparation

The sample preparation was performed according to the protocol described earlier^52^. Each bird lens was weighed prior to the homogenization: the typical lens weight for each bird species is shown in Table 1. Only one lens from each individual was used for the analysis. For grey heron (*Ardea cinerea*), only a half of the lens (with the mass of 175.1 mg) was taken for analysis. Each bird lens was homogenized with a TissueRuptor II homogenizer (Qiagen, Netherlands) in 1600 μL of cold (-20 °C) methanol, and then 800 μL of water and 1600 μL of cold chloroform were added. The mixture was shaken for 20 min and left at − 20 °C for 30 min. Then the mixture was centrifuged at 16,100×*g* and + 4 °C for 30 min, yielding two immiscible liquid layers separated by a protein layer. The upper aqueous layer (methanol–water) was collected and vacuum-dried for further analysis.

### NMR measurements

The extracts for the NMR measurements were dissolved in 600 μL of D_2_O containing 2 × 10^–5^ M of sodium 4,4-dimethyl-4-silapentane-1-sulfonic acid (DSS) as an internal standard and 20 mM of deuterated phosphate buffer (pH 7.1). ^1^H NMR measurements were carried out at the Center of Collective Use “Mass spectrometric investigations” SB RAS using a NMR spectrometer AVANCE III HD 700 MHz (Bruker BioSpin, Germany) as described in^52^. The concentrations of metabolites in the samples were determined by the peak area integration respectively to the internal standard DSS, and then recalculated into metabolite concentrations in the tissue (in nmoles per gram of the tissue wet weight).

### Optical measurements

For optical measurements, dry metabolomic extracts were dissolved in phosphate buffer (PBS). We obtained steady-state UV–Visible spectra using an Agilent 8453 spectrophotometer from Hewlett-Packard (La Jolla, CA, USA). Fluorescence measurements were performed with a FLSP920 (Edinburg Instruments, Edinburg, Great Britain) spectrofluorimeter. All measurements were carried out in a 10 × 10 mm^2^ quartz cell.

### Laser flash photolysis measurements

To study the properties of the excited states of NADH, nanosecond laser flash photolysis (LFP) measurements were performed. The experimental setup for LFP was described earlier^42^. Briefly, irradiation of the solution containing 0.2 mM of NADH in PBS (20 mM, pH 7.4) was carried out using a Quanta-Ray LAB-130–10 Nd:YAG laser from SpectraPhysics (Mountain View, CA, USA) at 355 nm. The detection was performed using a DKSh-150 xenon short-arc lamp from Stella Ltd. (Moscow, Russia). The solution was bubbled with argon for 15 min prior to and during irradiation.

### Steady-state photolysis

To measure the quantum yield of NADH photodegradation, 5 mM aqueous solution of NADH in a 10 × 10 mm^2^ quartz cell was irradiated using a DRSh-1000 mercury lamp. A water filter was used to cut off the infrared light and the spectral region of 300–400 nm was selected with a glass UV filter. Actinometry was performed according to the standard approach^53^ using the aqueous solution of potassium ferrioxalate. The photon flux at the surface of the quartz cell was (1.7 ± 0.2) × 10^18^ quanta per second. The solutions were bubbled with argon or oxygen for 15 min prior to and during irradiation. The total sample volume was 3 mL in each photolytic experiment. At certain time intervals the sample aliquots of 50 μL were taken for the further analysis by HPLC–MS and NMR. The total exposure time was 32 h for each photolytic experiment in argon and oxygen environments.

### LC–MS measurements

The LC–MS analysis was performed as described earlier^52^ at an UltiMate 3000RS chromatograph (Dionex, Germering, Germany) using a C18 Polar Advantage II (Dionex, Germering, Germany) column (3 × 150 mm, 3 μm). The detection was performed with an ESI-q-TOF high-resolution hybrid mass spectrometer maXis 4G (Bruker Daltonics, Bremen, Germany).

## Conclusions

This work is the first report on UV filters in the bird eye lens. We discovered that lenses of some raptors and waterfowl contain extraordinary high (up to millimolar) concentrations of NADH—a well-known coenzyme playing an important role in cellular redox reactions. In the bird eye, this compound acts as a UV filter, absorbing light in the UV-A spectral range, and, therefore, protecting the retina and the lens itself from the harmful effect of UV irradiation, and improving the visual acuity due to reducing the chromatic aberrations (especially achromatic vision). Photochemical properties of NADH meet all requirements for molecular UV filters: it features strong absorption in the UV-A spectral region, high photostability under both aerobic and anaerobic conditions, low yields of triplet state and radicals under irradiation, and the main channel of photoexcited state decay is the internal conversion to the ground state. In metabolomic measurements, we have not found evidence of the direct influence of high NADH concentration and low [NAD +]/[NADH] ratio on the metabolic processes inside the lens. The most likely explanation of this result is that active metabolic reactions in the lens are concentrated in the epithelial monolayer, and a large bulk of metabolically inert inner fiber cells conceals the possible metabolomic changes. The findings of this work contribute to our understanding of extremely high acuity of the bird vision, especially that of birds of prey.

## Supplementary Information

Supplementary Information.

## Data Availability

Raw NMR spectra, description of specimens and samples and metabolite concentrations are available at the Animal Metabolite Database repository, Experiment IDs 145, 199, and 218 (https://amdb.online/amdb/experiments/list/). All obtained data are available from the corresponding author upon request.
