# Supplementary material for: Nicotinamide adenine dinucleotide reduced (NADH) is a natural UV filter of certain bird lens
Source: Sci Rep. 2022 Oct 7;12:16850. doi: 10.1038/s41598-022-21139-x (PMC9546832; doi:10.1038/s41598-022-21139-x)

**Supplementary Information**

**for**

Acuity of the hawk eye: nicotinamide adenine dinucleotide reduced (NADH) is a natural UV filter of the bird lens

Nataliya A. Osik^1^, Ekaterina A. Zelentsova^1^, Kirill A. Sharshov^2^, Yuri P. Tsentalovich^1,*^

e-mail: [n.osik@tomo.nsc.ru](mailto:n.osik@tomo.nsc.ru), [zelentsova@tomo.nsc.ru](mailto:zelentsova@tomo.nsc.ru), sharshov@yandex.ru, [yura@tomo.nsc.ru](mailto:yura@tomo.nsc.ru)

^1^International Tomography Center SB RAS, Institutskaya 3a, Novosibirsk 630090, Russia

^2^Federal Research Center of Fundamental and Translational Medicine, Timakova str. 2, Novosibirsk 630117, Russia

* Corresponding author, e-mail: [yura@tomo.nsc.ru](mailto:yura@tomo.nsc.ru)

**Table of content**

Supplementary Table 1SI. Concentrations (mean ± std, in nmol per gram of wet tissue) of metabolites in lens tissues of bird species studied. ND – not detected. NQ – detected but not quantified.

Supplementary Figure 1SI. Hierarchical clustering analysis of lens metabolomic profiles of bird species performed with the use of Euclidian distance and Ward’s linkage.

Supplementary Table 1SI. Concentrations (mean ± std, in nmol per gram of wet tissue) of metabolites in lens tissues of bird species studied*. ND – not detected. NQ – detected but not quantified.

| **Metabolite** | *Milvus migrans*** | *Podiceps cristatus*** | | *Podiceps ruficollis* | *Podiceps auritus* | *Podiceps grisegena* | *Buteo buteo* | *Ardea cinerea* | *Larus ridibundus* | *Larus*  *vegae* | *Larus argentatus* | *Lanius excubitor* | *Circus aeruginosus* | *Columba*  *livia*** |
| --- | --- | --- | --- | --- | --- | --- | --- | --- | --- | --- | --- | --- | --- | --- |
|  | n=5 | n=5 | | n=1 | n=1 | n=1 | n=1 | n=1 | n=7 | n=3 | n=5 | n=1 | n=4 | n=12 |
|  | High [NADH] | | | | | | | Low [NADH] | | | | | | |
| **Proteinogenic amino acids** | | | | | | | | | | | | | | |
| Alanine | 1700 ± 400 | 2400 ± 1100 | | 3615 | 4510 | 3674 | 1120 | 1019 | 1200 ± 500 | 950 ± 100 | 1800 ± 900 | 1815 | 1700 ± 1200 | 1700 ± 600 |
| Asparagine | 170 ± 28 | 37 ± 17 | | 283 | 250 | 220 | 143 | 10 | 190 ± 80 | 92 ± 19 | 130 ± 40 | 210 | 121 ± 27 | NQ |
| Aspartate | ND | 220 ± 70 | | ND | 88 | 99 | 20 | 42 | 170 ± 70 | 128 ± 22 | 120 ± 60 | 95 | ND | 43 ± 21 |
| Glutamate | 790 ± 70 | 1020 ± 130 | | 915 | 1145 | 1292 | 558 | 926 | 1900 ± 800 | 1810 ± 70 | 1530 ± 220 | 3171 | 520 ± 130 | 2000 ± 300 |
| Glutamine | 1700 ± 300 | 4600 ± 1700 | | 6009 | 4481 | 3409 | 2534 | 4250 | 5200 ± 2400 | 3100 ± 400 | 4200 ± 700 | 10229 | 4000 ± 2300 | 3200 ± 500 |
| Glycine | 580 ± 110 | 900 ± 250 | | 881 | 1164 | 1693 | 415 | 894 | 800 ± 400 | 880 ± 100 | 970 ± 190 | 529 | 400 ± 90 | 350 ± 70 |
| Histidine | 120 ± 30 | 500 ± 100 | | 483 | 183 | 195 | 67 | 272 | 140 ± 70 | 110 ± 25 | 120 ± 70 | 153 | 180 ± 110 | 90 ± 30 |
| Isoleucine | 82 ± 20 | 77 ± 14 | | 65 | 172 | 170 | 118 | 51 | 44 ± 22 | 85 ± 5 | 60 ± 40 | 30 | 41 ± 11 | 36 ± 9 |
| Leucine | 190 ± 40 | 2200 ± 400 | | 1415 | 1563 | 2603 | 245 | 145 | 90 ± 40 | 151 ± 11 | 120 ± 70 | 106 | 180 ± 90 | 85 ± 13 |
| Lysine | 140 ± 40 | 910 ± 140 | | 433 | 619 | 662 | 122 | 306 | 70 ± 30 | 107 ± 9 | 46 ± 24 | ND | ND | 12 ± 30 |
| Methionine | 220 ± 50 | 350 ± 130 | | 569 | 250 | 303 | 170 | 46 | 280 ± 170 | 190 ± 50 | 160 ± 100 | 495 | 400 ± 300 | 200 ± 50 |
| Phenylalanine | 180 ± 70 | 53 ± 15 | | ND | 172 | 155 | 72 | 141 | 80 ± 50 | 105 ± 12 | 150 ± 90 | 68 | 130 ± 50 | 41 ± 16 |
| Proline | 480 ± 110 | 680 ± 240 | | 425 | 634 | 1132 | 692 | 440 | 700 ± 400 | 570 ± 130 | 510 ± 270 | 739 | 380 ± 160 | 600 ± 300 |
| Serine | 2670 ± 250 | 1190 ± 290 | | 1819 | 3420 | 2334 | 913 | 333 | 270 ± 160 | 179 ± 25 | 370 ± 250 | 4682 | 1700 ± 900 | 900 ± 240 |
| Threonine | 300 ± 80 | 860 ± 27 | | 760 | 791 | 1031 | 690 | 429 | 410 ± 170 | 430 ± 90 | 280 ± 90 | 346 | 900 ± 800 | 260 ± 100 |
| Tryptophan | 70 ± 40 | ND | | ND | 141 | 59 | 24 | 40 | 40 ± 30 | 67 ± 12 | 71 ± 25 | 74 | 25 ± 29 | ND |
| Tyrosine | 200 ± 50 | 125 ± 28 | | 138 | 417 | 509 | 138 | 311 | 150 ± 130 | 168 ± 18 | 190 ± 80 | 280 | 240 ± 120 | 140 ± 40 |
| Valine | 210 ± 30 | 186 ± 12 | | 172 | 371 | 286 | 321 | 135 | 120 ± 50 | 170 ± 5 | 140 ± 80 | 102 | 270 ± 170 | 107 ± 21 |
| **Other amino acids and amino acid derivatives** | | | | | | | | | | | | | | |
| Betaine | 590 ± 130 | 230 ± 100 | | 214 | 145 | 172 | 878 | 1080 | 1000 ± 400 | 1410 ± 290 | 1800 ± 500 | 1509 | 610 ± 150 | NQ |
| Citrulline | 1100 ± 500 | 6200 ± 1000 | | 3091 | 1068 | 492 | 355 | 3643 | 2600 ± 1200 | 2600 ± 400 | 1900 ± 1600 | ND | 1000 ± 210 | NQ |
| Creatine | 43 ± 10 | 52 ± 13 | | 90 | 3178 | 3188 | 37 | 871 | 90 ± 40 | 130 ± 40 | 160 ± 60 | 55 | 82 ± 15 | 27 ± 4 |
| Creatine phosphate | 24 ± 8 | 300 ± 70 | | 303 | 287 | 388 | 43 | 53 | 57 ± 26 | 44 ± 13 | 59 ± 27 | 76 | 19 ± 18 | NQ |
| Sarcosine | 7 ± 4 | 22 ± 20 | | 15 | 17 | 27 | ND | 196 | 90 ± 90 | 164 ± 10 | 190 ± 130 | ND | 11.1 ± 1.8 | 7 ± 4 |
| Taurine | 15000 ± 2900 | 3200 ± 500 | | 3108 | 7435 | 10176 | 20245 | 12239 | 9000 ± 4000 | 12200 ± 2300 | 12000 ± 4000 | 23322 | 15200 ± 1500 | 14500 ± 2100 |
| **Organic acids** | | | | | | | | | | | | | | |
| 2-hydroxy-3-methylbutyrate | 18 ± 3 | ND | | ND | 373 | 424 | 522 | 188 | 6.0 ± 2.8 | 15.4 ± 2.0 | 18 ± 9 | ND | ND | ND |
| 2-hydroxy-butyrate | 28 ± 12 | ND | | ND | 40 | 30 | 31 | 20 | 13 ± 6 | 14.5 ± 3.0 | 15 ± 3 | ND | 11 ± 13 | ND |
| 3-hydroxy-butyrate | 100 ± 50 | 170 ± 50 | | 197 | 459 | 267 | 296 | 185 | 280 ± 160 | 190 ± 40 | 90 ± 50 | 560 | 200 ± 80 | 90 ± 60 |
| 3-hydroxy-isovalerate | ND | 51 ± 12 | | 27 | 32 | 7 | 4 | ND | 14 ± 11 | 8.4 ± 1.8 | 16 ± 9 | 57 | 14 ± 3 | 190 ± 60 |
| Acetate | 4300 ± 1000 | 5100 ± 600 | | 534 | 300 | 193 | 121 | 78 | 170 ± 70 | 134 ± 14 | 170 ± 30 | 236 | 270 ± 50 | 89 ± 27 |
| α-Aminobutyrate | 63 ± 10 | 220 ± 90 | | 207 | 430 | 543 | 78 | 105 | 110 ± 50 | 50.5 ± 2.4 | 70 ± 30 | 78 | 280 ± 140 | 20 ± 6 |
| Formate | 75 ± 11 | 320 ± 60 | | 684 | 948 | 537 | 194 | 299 | 240 ± 100 | 147 ± 4 | 240 ± 30 | 921 | 290 ± 70 | 80 ± 50 |
| Fumarate | 13 ± 4 | 24 ± 6 | | 27 | 44 | 47 | 26 | 14 | 22 ± 9 | 27.0 ± 2.9 | 22 ± 5 | 30 | 31 ± 5 | 25 ± 7 |
| α-Hydroxyisobutyric acid | ND | ND | | ND | 10 | 12 | 18 | 6 | 4.5 ± 2.9 | 4.1 ± 1.2 | 6.1 ± 2.9 | 6 | 8 ± 7 | ND |
| Isobutyrate | 8 ± 5 | 10 ± 4 | | 17 | 8 | 16 | 18 | 10 | 13 ± 7 | 14 ± 5 | 10 ± 4 | 23 | 10 ± 4 | 1.5 ± 2.5 |
| Lactate | 13900 ± 2400 | 7700 ± 1600 | | 4100 | 11698 | 10730 | 9818 | 5090 | 8000 ± 4000 | 7700 ± 900 | 10000 ± 4000 | 6032 | 3920 ± 210 | 7100 ± 700 |
| Pyroglutamate | 270 ± 40 | 940 ± 180 | | 1704 | 1210 | 1132 | 173 | 1098 | 170 ± 70 | 119 ± 13 | 94 ± 17 | 898 | 380 ± 130 | 650 ± 120 |
| Pyruvate | 6.9 ± 1.4 | 11 ± 5 | | 19 | 8 | 11 | 12 | 52 | 6 ± 4 | 4.5 ± 1.8 | 9.8 ± 2.5 | 8 | 14 ± 3 | 12 ± 3 |
| Succinate | 23 ± 9 | 64 ± 11 | | 59 | 57 | 55 | 47 | 42 | 130 ± 60 | 119 ± 17 | 117 ± 22 | 62 | 35 ± 9 | NQ |
| **Metabolite** | *Milvus migrans*** | | *Podiceps cristatus*** | *Podiceps ruficollis* | *Podiceps auritus* | *Podiceps grisegena* | *Buteo buteo* | *Ardea cinerea* | *Larus ridibundus* | *Larus*  *vegae* | *Larus argentatus* | *Lanius excubitor* | *Circus aeruginosus* | *Columba*  *livia*** |
|  | n=5 | | n=5 | n=1 | n=1 | n=1 | n=1 | n=1 | n=7 | n=3 | n=5 | n=1 | n=4 | n=12 |
|  | High [NADH] | | | | | | | Low [NADH] | | | | | | |
| **Antioxidants** | | | | | | | | | | | | | | |
| Ascorbate | 390 ± 90 | 490 ± 70 | | ND | 216 | 296 | 560 | ND | 170 ± 80 | 200 ± 30 | 240 ± 80 | 480 | 430 ± 70 | 200 ± 40 |
| Ergothioneine | 1900 ± 800 | 9100 ± 1400 | | 5875 | 4492 | 2689 | 2270 | 3061 | 1800 ± 900 | 2200 ± 500 | 1300 ± 600 | 3585 | 2000 ± 500 | 1600 ± 300 |
| GSH | 1200 ± 500 | 6000 ± 1000 | | 5358 | 6080 | 7692 | 1024 | 49 | 1300 ± 500 | 1180 ± 110 | 730 ± 180 | 3118 | 1100 ± 400 | 2100 ± 400 |
| GSSG | 330 ± 120 | 350 ± 40 | | 800 | 487 | 316 | 127 | 1015 | 620 ± 180 | 530 ± 130 | 460 ± 140 | 210 | 130 ± 30 | NQ |
| **Alcohols, amines, sugars** | | | | | | | | | | | | | | |
| Choline | 54 ± 9 | 85 ± 15 | | 90 | 164 | 67 | 19 | 69 | 800 ± 500 | 730 ± 110 | 580 ± 170 | 30 | 52 ± 21 | 18 ± 7 |
| Glucose | 270 ± 200 | 500 ± 240 | | ND | 357 | 517 | 1341 | 1031 | 140 ± 130 | 260 ± 90 | 520 ± 280 | 1993 | 650 ± 250 | 1800 ± 500 |
| Gl-PhCholine | 53 ± 25 | 240 ± 120 | | 146 | 56 | 27 | 119 | 95 | 30 ± 14 | 80 ± 40 | 110 ± 70 | 355 | 71 ± 27 | NQ |
| Glycerol | 220 ± 80 | 240 ± 80 | | 176 | 338 | 161 | 146 | 184 | 150 ± 70 | 110 ± 40 | 121 ± 27 | ND | ND | ND |
| scyllo-Inositol | 50 ± 12 | 750 ± 250 | | 135 | 87 | 74 | 41 | 180 | NQ | NQ | NQ | ND | NQ | 55 ± 18 |
| myo-Inositol | 37600 ± 2100 | 27700 ± 3000 | | 27199 | 8652 | 4706 | 17639 | 33535 | 33000 ± 15000 | 42000 ± 3000 | 30000 ± 8000 | 15364 | 32000 ± 4000 | 37000 ± 4000 |
| PhCholine | 170 ± 50 | 114 ± 22 | | 105 | 149 | 227 | 194 | 185 | 270 ± 110 | 510 ± 130 | 610 ± 230 | 28 | 103 ± 14 | 250 ± 40 |
| **Nitrogenous bases, nucleosides, nucleotides** | | | | | | | | | | | | | | |
| ADP | 560 ± 80 | 720 ± 50 | | 670 | 336 | 112 | 183 | 104 | 430 ± 210 | 320 ± 50 | 320 ± 110 | 117 | 210 ± 270 | 470 ± 110 |
| AMP | 60 ± 40 | 51 ± 7 | | 38 | 55 | 17 | 13 | 13 | 60 ± 50 | 28 ± 13 | 24 ± 21 | ND | 24 ± 23 | 50 ± 50 |
| ATP | 2240 ± 290 | 3070 ± 280 | | 4019 | 4038 | 3573 | 3724 | 1046 | 3600 ± 1500 | 3590 ± 190 | 3000 ± 700 | 4222 | 4100 ± 700 | 3800 ± 500 |
| GTP | 120 ± 40 | 221 ± 26 | | 0 | 321 | 300 | 338 | 25 | 140 ± 60 | 230 ± 25 | 260 ± 140 | 151 | 150 ± 50 | 190 ± 70 |
| Hypoxanthine | 250 ± 40 | 125 ± 14 | | 119 | 141 | 107 | 163 | 191 | 70 ± 40 | 71 ± 17 | 57 ± 16 | 79 | 70 ± 25 | 185 ± 25 |
| Inosine | 89 ± 13 | 52 ± 14 | | 67 | 46 | 29 | ND | 16 | 27 ± 17 | 24 ± 8 | 10 ± 13 | ND | 37 ± 14 | 8 ± 14 |
| NAD | 500 ± 60 | 248 ± 28 | | 364 | 264 | 382 | 381 | 346 | 140 ± 60 | 154 ± 11 | 105 ± 23 | 140 | 187 ± 17 | 220 ± 40 |
| NADH | 1240 ± 80 | 560 ± 270 | | 982 | 1555 | 1624 | 254 | 135 | 40 ± 28 | 11 ± 3 | 12 ± 11 | ND | ND | 3 ± 5 |
| NADPH | ND | ND | | ND | ND | ND | ND | 113 | ND | ND | ND | ND | ND | ND |

* The individual metabolomic data for every species are available at the Animal Metabolite Database repository, Experiment IDs 145, 199, and 218 (https://amdb.online/amdb/experiments/list/).

** The data were reported in [18].

Supplementary Figure 1SI. Hierarchical clustering analysis of lens metabolomic profiles of bird species performed with the use of Euclidian distance and Ward’s linkage.


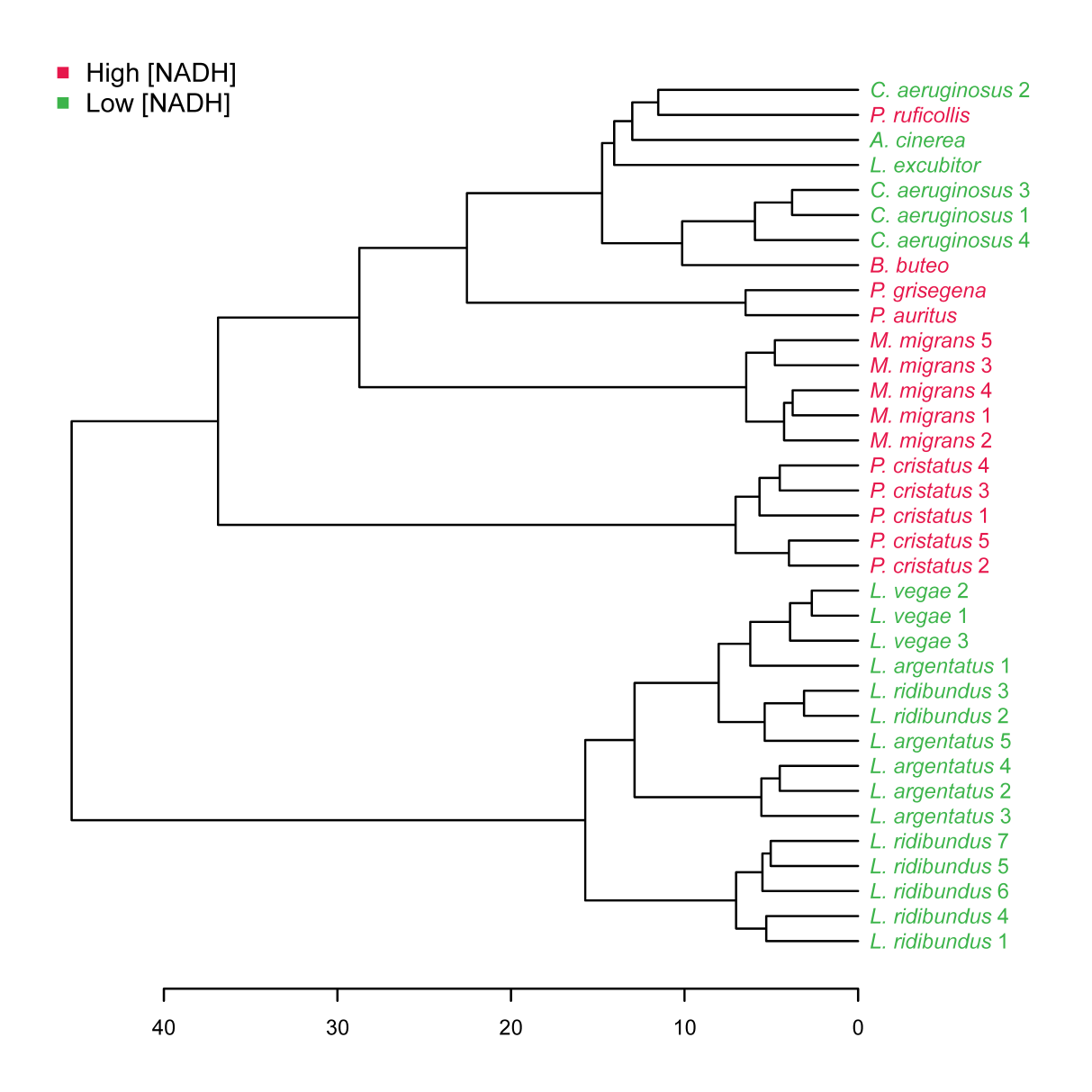

Supplement: Supplementary file 1 — Supplementary Information. [file 41598_2022_21139_MOESM1_ESM.docx]
